# The effect of environmental stressors on anti-mullerian hormone levels in Lebanese women: a retrospective study

**DOI:** 10.1371/journal.pone.0336016

**Published:** 2025-11-06

**Authors:** Sima Fatima Sharafeddin, Zeina Chehade, Samya El Sayed, Dima Salloum, Lara Nahouli, Antoine Hannoun, Ghina Ghazeeri

**Affiliations:** 1 Department of Obstetrics and Gynecology, American University of Beirut Medical Center, Beirut, Lebanon; 2 Faculty of Medicine, American University of Beirut, Beirut, Lebanon; 3 Division of Reproductive Science and Women’s Health Research, Department of Gynecology and Obstetrics, Johns Hopkins School of Medicine, Baltimore, Maryland, United States of America; Zhejiang University College of Life Sciences, CHINA

## Abstract

**Background:**

Prolonged and repetitive exposure to stressors leads to detrimental effects on female reproductive health; this can consequently increase susceptibility to stress and elevate risk of adverse mental health outcomes. Since October 2019, Lebanon has been engulfed in a multipronged crisis, beginning with a severe economic collapse, which was further exacerbated by the COVID-19 pandemic, followed by the catastrophic Beirut Blast in August 2020. These compounding events caused considerable stress, resulting in a substantial mental health burden. We employed a retrospective approach to trace the trend in Anti-Mullerian Hormone levels (the most widely used measure of functional ovarian reserve) in a sample of reproductive-aged Lebanese women in a tertiary healthcare center in Beirut in light of the events described.

**Methods:**

A retrospective chart review was performed of women aged 18–40 with AMH levels tested between January 2018 and June 2023 [excluding the time period between February 2020 – December 2020] (n = 563). Patients who had AMH levels between January 2018 – January 2020 were included in the ‘pre-stressful events’ group (n = 254) while those tested between January 2021 – June 2023 were in the ‘post-stressful events’ group (n = 283). Patient’s age, BMI, obstetrical and gynecological history, AMH level, and other relevant lab values were collected. Results were compared using the independent t-test.

**Results:**

Overall, this study demonstrated a lower mean AMH level in the post-stressful events group as compared to the pre-stressful events group, but this difference was not statistically significant. Patients with PCOS had a significant decline in the mean AMH level, as did patients with primary infertility.

**Conclusion:**

Women with pre-existing reproductive health disorders, such as PCOS and infertility, are more vulnerable to stress and are more likely to experience a decline in AMH level and, by extension, ovarian reserve following stressful experiences.

## Introduction

Fertility, the natural capacity to conceive and sustain a pregnancy, is a cornerstone of reproductive health and plays a vital role not only in individual well-being but also in family formation and broader demographic stability. Its significance extends beyond biological function, influencing psychological health, social identity, and even population dynamics, making the ability to preserve and assess fertility a priority in both clinical and public health contexts [1]. Infertility, defined as the inability to conceive after 12 months of unprotected intercourse, is estimated to affect between 8–12% of reproductive-aged couples worldwide [2]. Anti-mullerian hormone (AMH), a dimeric glycoprotein produced by the granulosa cells in ovarian follicles, is the most widely used measure of functional ovarian reserve [3]. In other words, AMH reflects the size of one’s remaining follicle pool, thereby serving as a proxy for reproductive capacity. It has shown clinical utility in predicting the success of in-vitro fertilization, diagnosis of polycystic ovary syndrome (PCOS) and premature ovarian failure (POF) [4]; elevated AMH level is a hallmark of PCOS, while decreased levels are a sensitive indicator of POF [5, 6]. More recently, AMH has been adopted in establishing a PCOS diagnosis [5], shown utility in the assessment of ovarian reserve in infertile patients, and assisted in guiding fertility care for women in this subgroup [7].

Fertility, and as a consequence AMH, are affected by multiple factors including medical, environmental and genetic factors. For example, AMH levels gradually decline with older age and are negatively affected by chemotherapy, history of ovarian surgery, use of oral contraceptive pills (OCPs), obesity, presence of BRCA mutation and possibly Vitamin D deficiency [8]. Given that stress has demonstrated negative effects on reproductive health and ovarian reserve, it follows that it adversely affects AMH levels [8].

Since October 2019, Lebanon has been engulfed in a multipronged crisis that has been cited as one of the top three most severe economic collapses since the mid-nineteenth century [9]. This has pushed more than 50% of the population into poverty and staggering inflation coupled with severe local currency depreciation that led to a tremendous curtailment of purchasing power. It has also had other devastating impacts; the resulting fuel shortages and inflation impacted access to essential services such as electricity, clean water, food, and healthcare [10]. The economic collapse was further exacerbated by political instability, the external shock of COVID-19 pandemic, and the catastrophic Port of Beirut explosion in August of 2020 [10,11]. These compounding national and international events caused considerable stress, resulting in a substantial mental health burden. Elbejjani et. al assessed the mental health challenges of the Lebanese population during the first COVID-19 lockdown using multiple validated scales, and reported depressive and anxiety symptoms as being among the highest worldwide [12]. Furthermore, deteriorating living conditions were further exacerbated by the 2020 Beirut blast which occurred in a densely populated area of the city leaving thousands injured [13], and approximately 218 dead [14]. In a survey of 2078 survivors of the blast, one-third met the criteria for post-traumatic stress disorder, and 80% screened positive for depression. Notably, women of lower socioeconomic status who were closest to the site of the explosion were most affected [15].

A stressor is defined as any physical or psychological stimulus that leads to physiological or behavioral changes (also known as stress response) mediated by the hypothalamic-pituitary-adrenal (HPA) axis and the sympathetic-adrenal-medullary (SAM) axis [16]. Prolonged and repetitive exposure to stressors results in lasting neuroendocrine changes leading to detrimental effects on female reproductive health (including ovarian reserve, fertility, and fecundity) [17]. Increasing concentrations of glucocorticoids (reaching ‘stress’ levels) lead to direct inhibition of the hypothalamic release of gonadotropin-releasing hormone (GnRH), consequently disrupting the hypothalamic-pituitary-ovarian (HPO) axis [18]. Animal studies have demonstrated that stress levels of glucocorticoids prevent an increase in estrogen secretion and the subsequent luteinizing hormone (LH) surge, negatively impacting follicular maturation and release, as reported in a sheep model [19]. Chronic psychological stress resulted in the downregulation of AMH protein expression in mouse ovaries [20]. Higher levels of perceived psychosocial stress led to lower levels of AMH in infertile [21] women, sub-fertile [22] women, and female childhood cancer survivors [23].

In turn, some reproductive health disorders may themselves increase susceptibility to stress and elevate the risk of adverse mental health outcomes. For instance, 17% of women with PCOS have a diagnosis of depression, and 41% report anxiety symptoms [24]. Similarly, many patients with infertility experience anxiety and depressive symptoms, with the prevalence of depression among them reported to be as high as 52% [25]. Premature ovarian insufficiency (POI) is also associated with vulnerability to stress with higher odds of depression [OR: 3.33] and anxiety [OR: 4.89] [26].

Research on stress in the context of reproductive outcomes recognizes multiple measures of ‘stress’, including exposure to acute stressful events, living in stressful environments, or heightened susceptibility to stress [27]. In a cross-sectional study of infertile women, exposure to chronic lifetime psychosocial stressors (such as history of abuse or substance use) was more strongly associated with a decline in ovarian reserve relative to increased ‘current stress’ levels [28]. This underscores the need for careful evaluation of onset, chronicity, and pattern of stress exposure to better contextualize and measure its effect on reproductive health outcomes.

Comprehensive national studies specifically tracking AMH levels in Lebanon since 2019 are lacking. As an alternative, fertility clinics and reproductive health centers often conduct their own assessments to provide individualized treatment plans. As such, this study employs a retrospective approach to trace the trend in AMH levels in a population of reproductive-aged Lebanese women in a tertiary healthcare center in the country’s capital, Beirut, in the setting of compounding national and international events that caused significant disruptions to daily life.

## Materials and methods

### Design and setting of the study

Ethical approval was obtained from the American University of Beirut Institutional Review Board (IRB) prior to conducting the study (IRB ID BIO-2023–0229). Medical records of patients at the American University of Beirut Medical Center were retrospectively reviewed via the electronic medical record system. The records reviewed were taken from the period January 1, 2018, until June 30, 2023; the period of stressful events was defined as the period from February 1, 2020, to December 1, 2020. Medical records with AMH levels taken during this period were excluded to better homogenize the post-stress group, enable a more accurate comparison of AMH levels, and eliminate potential bias from the stress itself.

Patients between the ages of 18 and 40 years, with AMH levels tested at the American University of Beirut Medical Center and available on their medical record within the specified time period were included. Women outside the specified age range, those with known hormonal disorders, and women undergoing active treatment for malignancy at the time the AMH level was taken were excluded.

Since this study was a retrospective chart review, the data was pre-existing, and no patients were contacted for information nor was there new data collected prospectively. As such, consent to participate was not required as per the requirements of the institutional review board at the American University of Beirut, and a waiver for patient consent was granted prior to conducting the study.

### Participants

A sample of 563 patient records were initially reviewed by the authors from November 15, 2023 until February 29, 2024. Of the original 563, 26 subjects were excluded due to having a history of hormonal disorders or were receiving treatment for hormonal disorders. The final number of records reviewed and included in the analysis was 537, with 254 subjects enrolled in the control (pre-stressful events) group and 283 enrolled in the exposure (post-stressful events) group. Authors accessing the medical records were able to view information that could identify individual participants during data collection.

### Measures

Patients’ results for serum AMH, Follicle Stimulating Hormone (FSH), LH, Estradiol, Prolactin, Thyroid Stimulating Hormone (TSH), and Vitamin D levels were collected to assess baseline hormonal measures. AMH levels were measured using electrochemiluminescence immunoassay (ECLIA).

Relevant obstetrical and gynecological history was retrieved. This included the patients’ pregnancy history, history of infertility, history of assisted reproductive technology (ART), and history of gynecological pathologies such as endometriosis, PCOS, and POI. Any history of intrauterine insemination, in vitro fertilization, or intracytoplasmic sperm injection was considered as a positive ART history.

Confounding factors affecting the gynecological history such as age, BMI, personal or family history of POF were also collected.

### Statistical analysis

The results were calculated using SPSS version 24 statistical software package (IBM, USA). Demographic characteristics were summarized using descriptive statistics. Independent samples *t*-tests were performed to confirm that the pre- and post-stressful events groups were comparable with respect to demographic variables.

Independent t-test was carried out to assess the differences in AMH means between the pre- and post-stressful events groups. Specific confounding factors that could affect AMH level, including PCOS, POF, diminished ovarian reserve (DOR), and a diagnosis of infertility were controlled for and examined via independent t-test to evaluate their effect on the results of each group both individually and collectively. Given multiple comparisons, we applied a Bonferroni correction to adjust the significance threshold to *p* < 0.0125.

## Results

Table 1 depicts subjects’ demographic information such as age and marital status, as well as baseline lab values. Both groups exhibited homogeneity in terms of demographic information and baseline laboratory values, rendering them comparable.

**Table 1:** Overview of subject demographics.

	Pre-stressful events	Post-stressful events	P value
**Demographics**			
BMI (kg/m^2^)	24.28 ± 4.11	24.48 ± 4.33	0.59
Age (years)	32.08 ± 5.2	32.82 ± 5.25	0.1
**Marital Status**			
Single	72 (28.3%)	104 (36.7%)	
Married	181 (71.3%)	171 (60.4%)	
Divorced	1 (0.4%)	8 (2.8%)	0.17
**Smoking frequency**			
Never	166 (66.9%)	159 (56.8%)	
Occasionally	13 (5.2%)	23 (8.2%)	
Some days	11 (4.4%)	24 (8.6%)	
Daily	42 (16.9%)	57 (20.4%)	
Former	16 (6.5%)	17 (6.1%)	0.26
**Lab Values**			
FSH (mIU/mL)	8.75 ± 9.47	8.99 ± 13.64	0.87
LH (mIU/mL)	8.64 ± 7.11	8.34 ± 9.03	0.79
Estradiol (pg/mL)	67.7 ± 79.16	81.32 ± 109.01	0.27
Prolactin (ng/mL)	14.82 ± 9.87	16.47 ± 8.85	0.13
TSH (µU/mL)	1.81 ± 0.92	1.74 ± 0.95	0.55
Vitamin D (ng/mL)	23.05 ± 10.83	25.2 ± 12.68	0.18

The significance of differences in mean AMH levels before and after stressful events, between different age groups, and in patients with any history of receiving assisted reproductive technology is shown in Table 2. The mean AMH level in individuals who had not experienced the stressful events (2.52 ± 2.94 ng/mL) was slightly greater than that in those with exposure to all three stressful events (2.12 ± 1.99 ng/mL), however the p-value was not significantly different (p = 0.061).

**Table 2:** Independent T-test comparing AMH levels by group, age, and history of assisted reproduction.

	AMH (mean ± SD)	P-value
**Time AMH was taken (N)**		
Pre-stressful events (254)	2.52 ± 2.94	
Post- stressful events (283)	2.12 ± 1.99	0.061
**Age group (N)**		
Below 35 years old (351)	2.66 ± 2.75	
36–40 years old (186)	1.64 ± 1.74	<0.01*
**History of ART (N)**		
Yes (110)	1.76 ± 1.82	
No (427)	2.446 ± 2.62	0.011*

*P-value<0.05 was considered significant

When stratified by age, participants below 35 years old had significantly higher AMH levels (2.66 ± 2.75 ng/mL) compared to those aged 36–40 years old (1.64 ± 1.74 ng/mL, p-value < 0.01). Furthermore, individuals with a history of ART exhibited significantly lower AMH levels (1.76 ± 1.82 ng/mL) compared to those without such history (2.45 ± 2.62 ng/mL, p = 0.011).

**Fig 1:**
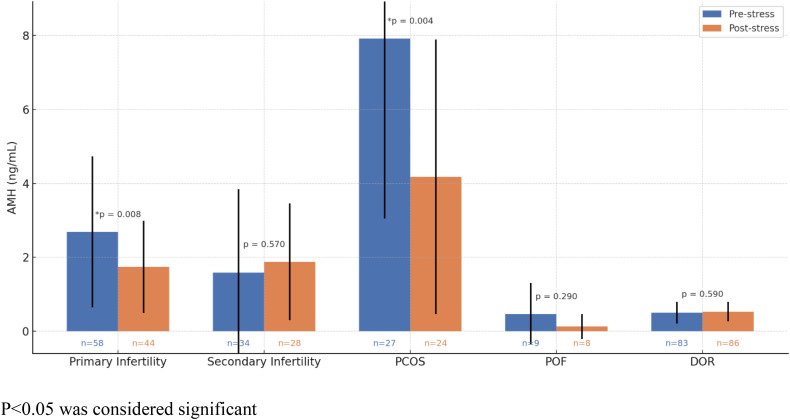
Controlling for confounding actors. P < 0.05 was considered significant.

Although patients with hormonal disorders such as hypothyroidism and hyperprolactinemia were excluded at the start of the study, patients with PCOS and DOR were not. A diagnosis of PCOS is established when an individual exhibits at least two of the following criteria: clinical or biochemical hyperandrogenism, polycystic appearance of ovaries, and oligo-anovulation [27]. Fig 1 depicts the secondary analysis done to control for the effect of PCOS, DOR, POF, and infertility on AMH levels within the sample. When controlling for PCOS cases, a significant difference in AMH levels between the two groups was observed (p = 0.004), where the mean AMH in the pre-stressful events group (7.92 ± 4.87) was nearly twice as much as that of the post-stressful events group (4.18 ± 3.71). These findings remained significant after Bonferroni correction (adjusted α = 0.0125). Regarding subjects with DOR and POF, both groups did not yield significant results when controlled for (p = 0.59; p = 0.29 respectively).

Among subjects with primary infertility, the mean AMH was significantly higher pre-stressful events (2.69 ± 2.04 compared to 1.74 ± 1.25 post-stressful events; p = 0.008), while subjects with secondary infertility showed no significant differences in mean AMH level (1.59 ± 2.25 in the pre-stressful events group compared to 1.88 ± 1.58 post-stressful events; p = 0.57).

**Fig 2:**
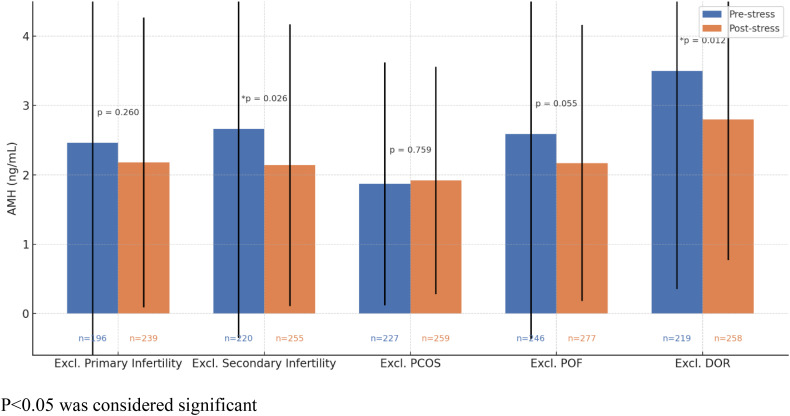
Excluding confounding actors. P < 0.05 was considered significant.

Fig 2 focuses on comparisons of AMH levels after excluding subgroups from the entire sample. Excluding DOR showed a significant decrease in AMH level post-stressful events (p = 0.012) after Bonferroni correction (adjusted α = 0.0125). After excluding subjects with secondary infertility from the sample, a decline in AMH levels is observed after stressful events (from 2.66 ± 3.01 to 2.14 ± 2.03, p = 0.026), though it is not considered significant after adjusting the level of significance (adjusted α = 0.0125). The other subgroups did not exhibit significant changes in mean AMH levels, further verifying the results displayed in Fig 1.

## Discussion

Various studies have suggested a link between psychological stress and decreased AMH levels in women of reproductive age. This retrospective study demonstrated lower levels of AMH in subjects exposed to Lebanon’s multipronged crisis compared to those not exposed to such stressors. Notably, statistically significant differences were found only in women with primary infertility and polycystic ovarian syndrome.

The existing literature shows conflicting data regarding the effect of stress on AMH levels. For instance, Lawal et al. reported no correlation between self-reported stress and psychological distress and serum anti-Müllerian hormone levels in infertile and fertile women of reproductive age [29]. In contrast, Dong YZ et al. assessing the impact of stress on AMH levels showed that psychological stress is associated with lower AMH levels [21]. Similarly, Yeğin et al. also demonstrated that healthcare professionals during the recent COVID-19 pandemic exhibited low AMH levels, where lower levels were related to anxiety severity [30].

The main difference arising among these studies may lie in the type and intensity of the stressor. Acute stress is a short-term response to an immediate threat, typically triggering a “fight or flight” reaction. When stress becomes chronic, sustained HPA axis activation disrupts the balance between cortisol, gonadotropins, and ovarian hormones [31]. This dysregulation may impair granulosa cell function and follicular development and may result in the loss of growing follicles due to the oxidative damage, thereby reducing AMH production and resulting in a decrease in AMH levels [21]. The heterogeneity of stressors studied—ranging from everyday stress to pandemic-related trauma—likely contributes to the variability in reported results [21,29,30]. The heterogeneity of stressors might have led to conflicting results in the literature.

Another factor to consider is the individualized response to stress among those exposed; there appears to be a genetic role in one’s response to trauma, where the same exposure can elicit different responses in different individuals [32].

Lifestyle factors (e.g., diet, exercise) [33] and pre-existing psychological conditions (e.g., anxiety or depression) can also serve as confounding variables to AMH level fluctuations. Data regarding the association of specific psychiatric conditions such as anxiety and depression with lower AMH levels has been conflicting. While Jeon et al. suggested that higher depressive scores were associated with lower serum AMH levels [34], and Yeğin et al. concluded that higher anxiety severity among healthcare professionals during COVID-19 was associated with lower AMH levels [30], Golenbock et al.’s cross sectional study found that there was no association between a history of depression and lower AMH levels [35].

To account for confounding factors in our study, a secondary analysis was performed where PCOS, POF and DOR cases were both controlled for and excluded from the dataset. Cases of primary and secondary infertility were also controlled for and excluded for the secondary analysis.

Controlling for PCOS cases displayed a decline in AMH levels by almost half after stressful events, which was statistically significant. This corroborates Park et al.‘s findings that women with PCOS exhibit heightened susceptibility to emotional stress, with serum AMH levels inversely related to depression and stress in these patients [36]. Damone et al. also demonstrated that women with PCOS have increased anxiety, depression, and perceived stress [37]. Psychological distress could have led to drastic hormonal changes in this group within our sample, and a subsequent decline in AMH levels. To our knowledge, there are no available studies exploring the impact of stress on AMH levels in patients with PCOS. On the contrary, in the study by Yeğin et al., individuals with PCOS were deliberately omitted from the analysis [30].

When excluding PCOS or POF cases from our dataset for secondary analysis, the difference between both groups’ mean AMH levels remained statistically insignificant. The significance of lower AMH levels after a stressful event was only observed when excluding cases of diminished ovarian reserve; this suggests that including subjects with DOR in the original sample may have masked the significant decrease in AMH levels following the occurrence of stressful events. Furthermore, extremes of variability in levels of AMH may exhibit disparities among these subcategories, potentially influencing the variations observed in subgroup analysis as well as the lack of significance noted in the primary analysis.

When controlling for cases of primary and secondary infertility from our dataset, a statistically notable drop in AMH level was demonstrated among patients with primary infertility following stressful events, while no statistical difference could be noted in patients with secondary infertility. This finding aligns with the results of Greil et al., which showed that patients with primary infertility stand out as a particularly distressed group compared to patients who already had a previous child [38]. This situation places them in a more vulnerable position when facing additional life stressors [39].

In order to examine potential confounding factors of the results, subjects’ age, BMI and smoking status were assessed. This study aligned with existing literature regarding age and AMH, where participants under 35 years old had significantly higher AMH levels compared to those aged 36–40 years old. Although the decline trajectory of AMH is not uniform in all women [38], serum AMH level is the current best endocrine marker to assess the age-related decline in fertility [40,41].

Although previous studies have identified BMI and smoking status as factors affecting AMH levels, the literature on this topic remains mixed, with some studies reporting negative associations between BMI or smoking and AMH [42–44], while others have found no clear relationship [45–47]. Our analysis did not reveal significant associations between either variable and AMH levels in our cohort. While this may reflect a true absence of effect in our sample, it is also possible that the lack of significance is due to population-specific factors, sample size, or the influence of other, unmeasured variables.

Other studies examining lifestyle factors affecting AMH levels primarily focused on the effect of diet and exercise [48,49]. However, these studies examined subjects with other pre-existing factors influencing AMH levels, such as PCOS [49] and obesity [50,51]. Since this study was retrospective, it was not feasible to assess lifestyle factors which might influence AMH serum levels, such as diet, exercise, or hours of sleep.

It is difficult to assess the role of genetics and hereditary factors on one’s AMH levels, given the fluctuating nature of the hormone which is heavily influenced by environmental factors. A review of the existing literature on AMH levels as a measure of functional ovarian reserve done by Moolhuijsen and Visser [3] concluded that while AMH remains the preferred marker for ovarian reserve, interpretation of serum levels is limited due to a lack of understanding of the genetic and lifestyle factors influencing AMH levels. Studies examining AMH levels as a hereditary factor largely focused on the utility of AMH levels as a predictor of menopausal age [52–54]. Studies on the effect of genetics and familial patterns on AMH serum levels are lacking.

Individuals with a history of ART exhibited significantly lower AMH levels compared to those without such history; however, the reason behind the need of ART was not mentioned nor assessed, and originally having low AMH level could be the indication for ART rather than the consequence. Furthermore, this result could be due to the large difference in the number of subjects (n = 427 for those who have not done ART, compared to n = 110 for those who have).

To our knowledge, our study is the first to explore AMH levels in connection to three major catastrophic events in the past four years in Lebanon, particularly in patients with PCOS and primary infertility. Our study was conducted with a large sample size to analyze stress effects on various subgroups, leading to a general conclusion.

Our investigation encountered several limitations. Primarily, it was retrospective in nature, and potential selection bias was not mitigated due to the absence of an objective measure for stress impact on participants. Additionally, assumptions regarding the post-stress group’s exposure to stressors were made solely based on recorded AMH test dates, with no direct contact to verify their experiences or to consider confounding factors such as pre-existing mental health conditions, lifestyle factors, and socioeconomic status. Furthermore, factors affecting AMH levels such as oral contraception use and prior ovarian surgery were not taken into account during the study, which could affect the study’s results.

## Conclusion

Women with pre-existing reproductive health conditions experience a higher vulnerability to stressors, such as women with PCOS and primary infertility. Individuals with such conditions are more likely to experience significant declines in AMH levels and by extension, ovarian reserve following stressful events. These findings highlight the need for a holistic approach to reproductive health, one that considers both physical and emotional factors to better understand and support fertility outcomes.
